# Design and Preclinical Evaluation of Chitosan/Kaolin Nanocomposites with Enhanced Hemostatic Efficiency

**DOI:** 10.3390/md19020050

**Published:** 2021-01-22

**Authors:** Mahmoud Elsabahy, Mostafa A. Hamad

**Affiliations:** 1 Science Academy, Badr University in Cairo (BUC), Cairo 11829, Egypt; 2 Science Park, Misr University for Science and Technology, Giza 12566, Egypt; 3 Department of Surgery, Faculty of Medicine, Assiut University, Assiut 71515, Egypt

**Keywords:** chitosan, kaolin, hemostatic, dressings, in vivo, nanocomposites, QuikClot Combat Gauze

## Abstract

In the current study, hemostatic compositions including a combination of chitosan and kaolin have been developed. Chitosan is a marine polysaccharide derived from chitins, a structural component in the shells of crustaceans. Both chitosan and kaolin have the ability to mediate a quick and efficient hemostatic effect following immediate application to injury sites, and thus they have been widely exploited in manufacturing of hemostatic composites. By combining more than one hemostatic agent (i.e., chitosan and kaolin) that act via more than one mechanism, and by utilizing different nanotechnology-based approaches to enhance the surface areas, the capability of the dressing to control bleeding was improved, in terms of amount of blood loss and time to hemostasis. The nanotechnology-based approaches utilized to enhance the effective surface area of the hemostatic agents included the use of Pluronic nanoparticles, and deposition of chitosan micro- and nano-fibers onto the carrier. The developed composites effectively controlled bleeding and significantly improved hemostasis and survival rates in two animal models, rats and rabbits, compared to conventional dressings and QuikClot^®^ Combat Gauze. The composites were well-tolerated as demonstrated by their in vivo biocompatibility and absence of clinical and biochemical changes in the laboratory animals after application of the dressings.

## 1. Introduction

Injury is a major public health problem that is considered as the leading cause of death among persons in the age between 1–44 years in the USA, according to the Centers for Disease Control and Prevention (www.cdc.gov/injury). It is also responsible for more than 5 million deaths per year, with an estimated cost of $518 billion globally (www.aast.org/resources/trauma-facts). In trauma patients, hemorrhage is the second leading cause of deaths preceded only by central nervous system injury. Hemorrhage constitutes up to 40% of trauma mortality, of which 33–56% occur during the prehospital period [1]. Hemorrhage may be also encountered during surgical procedures.

Hemostatic dressings are regularly applied early after hemorrhage, irrespective of the cause, to reduce blood loss, until the patients receive the necessary medical care in the appropriate health care units. Currently, there are several hemostatic dressings that are either in the market or under development [2,3,4,5]. These dressing could be classified into polysaccharide-based (e.g., chitin/chitosan), zeolite/kaolin-based, dry fibrin sealant, microporous polysaccharide hemospheres, and hydrophilic polymers of potassium salts. For example, kaolin is the active hemostatic agent in QuikClot^®^ Combat Gauze that is the hemostatic dressing of choice used on the battlefield [6,7,8]. Kaolin is aluminum silicate that activates the intrinsic pathway of coagulation via concentrating clotting factors through the rapid absorption of the water content of blood at the bleeding site. Other commercially available hemostatic dressings include Celox™ gauze and Hemcon^®^, which both depend on including chitosan as the hemostatic agent [9,10]. Chitosan is a naturally occurring biodegradable polysaccharide that breaks down in the body into glucosamine and *N*-acetyl glucosamine. It is obtained through the partial deacetylation of chitin in the shells of shrimp and other crustaceans. Chitosan is extensively used in food and pharmaceutical industries. It has intrinsic bioadhesive and antimicrobial properties and it induces hemostasis via electrostatic interactions with the negatively charged cell membranes of the red blood cells (RBCs), and, thus strongly adhering to and sealing the bleeding site [11,12,13].

Although commercially available hemostatic agents can be effective in stopping bleeding of mild hemorrhages, improved hemostatic dressings are needed to control severe bleeding (e.g., in the battlefield). We have previously designed chitosan-loaded β-cyclodextrin polyester hydrogels to serve as degradable absorbable composites that can be left in the injury site and degrade to reduce the duration of interventional procedures [14]. Assembly of chitosan into nanofibrous honeycomb-like mats with enhanced surface area resulted in improved hemostatic efficiency in vivo in acute liver injury animal models. However, the absorbable composites might be more appropriate to control mild-to-moderate bleeding that is encountered, for example, during intraabdominal surgeries. For severe injury (e.g., femoral artery and veins injuries), the use of robust hemostatic systems could be more appropriate [15]. 

The current study aims at providing a hemostatic dressing of an improved efficiency in controlling severe bleeding from external and internal wounds. The hemostatic composition includes two hemostatic agents (chitosan and kaolin), in combination with a surfactant. The surfactant is included in the composition to enhance disbursement and increase the surface area of kaolin. Alternatively, deposition of chitosan micro- and nano-fibers could also improve hemostatic efficiency via enhancing the surface area of the hemostatic agents utilized. In the current study, the term “surface area” represents an approximate measure of the total area of the carrier that is covered or occupied by the hemostatic agents. The developed composites could significantly reduce bleeding to a greater extent compared to dressings lacking a nanoparticularized hemostatic agent and/or utilizing a single hemostatic agent. 

## 2. Results

### 2.1. Preparation of Chitosan/Kaolin Nanocomposites of Enhanced Surface Area

**Figure 1:**
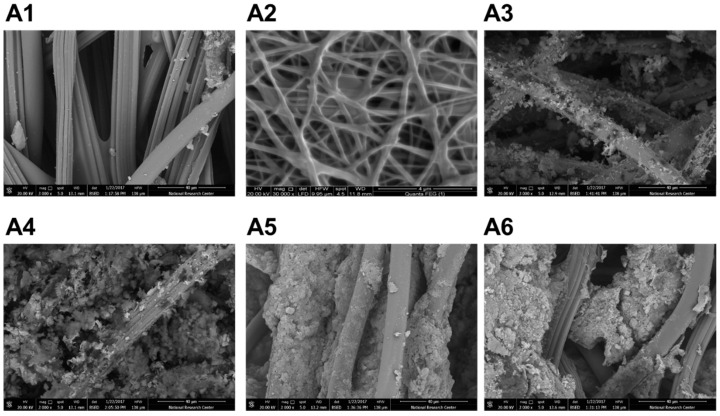
Field Emission scanning electron microscopy images that demonstrate the increase in surface areas of the various hemostatic agents used either by converting the chitosan into microfibers (**A1**, 3000×) or nanofibers (**A2**, 30,000×), or by modifying the conventional method of kaolin impregnation that usually provides inefficient coating onto the carrier (**A3**, 3000×) to a more efficient coating via the use of Pluronic nanoparticles (**A4**, 3000×) and chitosan microfibers (**A5**,**A6**, 3000×).

Hemostatic composites were designed to achieve prompt and efficient hemostasis via increasing the specific surface area of one or more hemostatic agents, to increase the coating efficiency onto the carrier (i.e., gauze) and the contact with the body tissues and blood cells. Several approaches were utilized and potential synergistic hemostatic effect between hemostatic agents was also explored. However, only composites that have demonstrated significant hemostatic efficiency were systematically studied. Of the effective approaches, was to convert chitosan (i.e., hemostatic agent) into micro- or nano-based fibers, via deposition on conventional gauze or through electrospinning, respectively. Chitosan microfibers were formed spontaneously upon deposition onto the carrier (gauze). Throughout the manuscript, the term “nanofibers” refers to electrospun chitosan/polyvinyl alcohol (PVA) solution at a ratio of 50/50. It can be seen how chitosan micro- and nano-fibers could result in high surface area and efficient coating onto the carriers (Figure 1A1,A2). The size of the gauze threads without any coating was 14.6 ± 2.4 µm. The chitosan micro- and nano-based fibers had diameters of 2.4 ± 0.9 µm and 101 ± 56 nm, respectively. Another example of hemostatic agents used is kaolin. The use of heated kaolin slurry in coating non-woven gauze is the most commonly utilized method to prepare hemostatic dressings [16,17,18]. However, this method results in low coating efficiency as can be seen in the figure (Figure 1A3), which explains the relatively low hemostatic efficiency of this kind of preparations (vide infra). On the contrary, the use of pre-assembled Pluronic nanoparticles (Pluronic^®^ F-127) (Figure 1A4) or chitosan microfibers (Figure 1A5,A6) to disperse the kaolin particles resulted in high coating efficiency onto the gauze. Kaolin alone and when dispersed with the assistance of Pluronic micelles had diameters of 7.9 ± 2.2 µm and 4.7 ± 1.9 µm, respectively. When the dispersion process was assisted with deposition of chitosan, in the presence or absence of glycerin, kaolin particles had diameters of 4.2 ± 2.6 µm and 3.9 ± 1.5 µm, respectively. The size of kaolin particles did not change significantly, but rather their dispersibility and coating efficiency onto the carriers had greatly increased (Figure 1). It can be overall concluded that the use of nano- or micro-technology-based approaches aids in enhancing surface areas of hemostatic agents and in improving the coating efficiency onto the carrier used (i.e., gauze). 

### 2.2. In Vivo Assessment

The research protocol was revised and approved by “the Committee of Medical Ethics” of the Faculty of Medicine in Assiut University, Assiut, Egypt. All animals received care and were used in strict compliance with the Guide for the Care and Use of Laboratory Animals.

#### 2.2.1. In Vivo Hemostatic Efficiency in Rabbits

The hemostatic efficiency was tested in two animal models: Rabbits and rats. The animal models were developed by cutting the femoral artery and vein, and applying immediately the hemostatic agents under pressure (weight of 200 g for 3 min) and measuring the survival rates, survival durations, total amount of blood loss, time to hemostasis, and mean arterial blood pressure (MAP). Dressings were applied to the injury sites for a duration of 30 s per application, until hemostasis is achieved. Active bleeding was generally examined every 30 s. No fluid resuscitation or surgical intervention was allowed during the experimental setup. The weights of the dressings were recorded before application and at the end of the experiments to measure the amounts of blood loss. MAP was recorded via insertion of a 20-gauge catheter into the right carotid artery and continuous recording of MAP under general anesthesia before the injury and for 1 h after the injury. 

**Figure 2:**
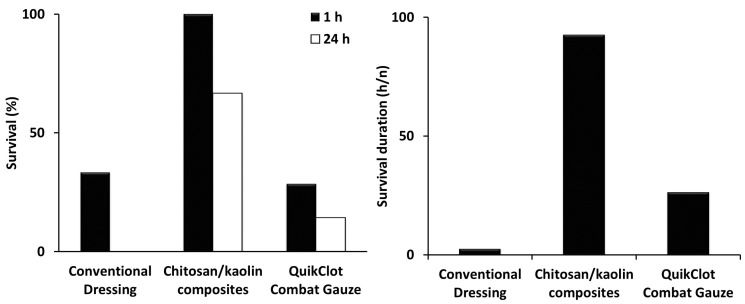
(**Left**): Percentages of survival in rabbits, 1 h and 24 h, after cutting their femoral arteries and veins. (**Right**): The mean survival durations (total number of survival hours/number of animals in each group). Conventional dressings, chitosan/Kaolin composites, and QuikClot^®^ Combat Gauze were applied immediately after injury with immediate compression for 3 min. The kaolin/chitosan composites resulted in significantly longer survival duration compared to the conventional dressing (*p* < 0.01) and QuikClot^®^ Combat Gauze (*p* < 0.05). The difference between the conventional dressing and QuikClot^®^ Combat Gauze was not statistically significant.

**Figure 3:**
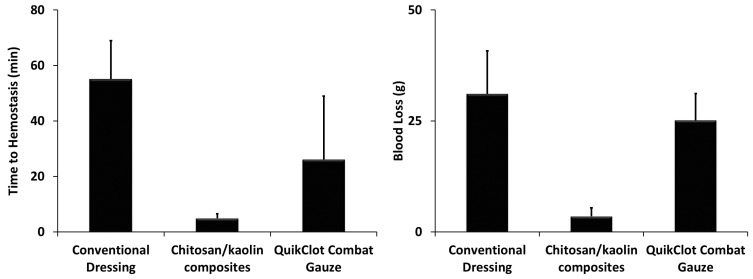
(**Left**): Total time to hemostasis in rabbits after cutting their femoral arteries and veins. Measurements were performed over one hour and rabbits that died were assigned to a time to hemostasis of 60 min. The kaolin/chitosan composites resulted in significantly faster time to hemostasis compared to the conventional dressing (*p* < 0.0001) and QuikClot^®^ Combat Gauze (*p* < 0.05). The difference between conventional dressing and QuikClot^®^ Combat Gauze was also statistically significant (*p* < 0.01). (**Right**): Total amounts of blood loss were evaluated by absorbing the leaked blood into conventional gauze, and the differences in the total weight of all the used pieces before and after the experiments were calculated. The kaolin/chitosan composites resulted in significantly lower amounts of blood loss compared to the conventional dressing (*p* < 0.0001) and QuikClot^®^ Combat Gauze (*p* < 0.0001). The difference between conventional dressing and QuikClot^®^ Combat Gauze was not statistically significant. In both experiments, dressings were applied immediately after injury with immediate compression for 3 min.

**Figure 4:**
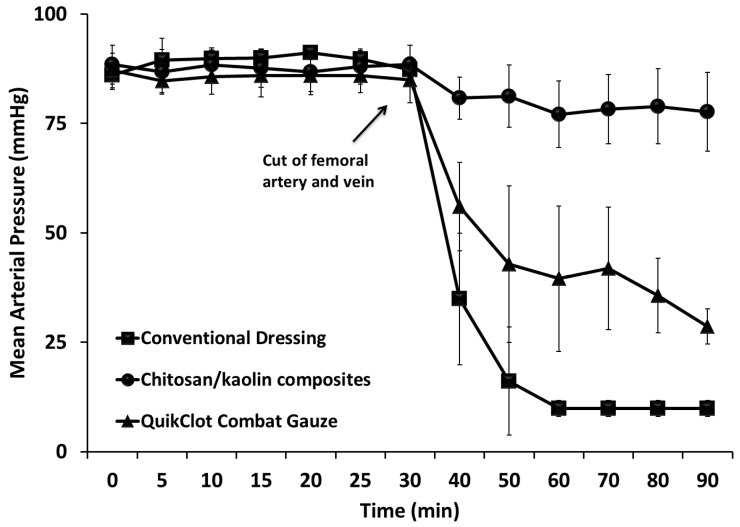
The mean arterial pressure of rabbits after cutting their femoral arteries and veins. Conventional dressings, chitosan/Kaolin composites, and QuikClot^®^ Combat Gauze were applied immediately after injury with immediate compression for 3 min. The measurements over the first 30 min represent the mean arterial pressure of rabbits before cutting their femoral arteries and veins.

The dressings that comprised one hemostatic agent have demonstrated poor hemostatic efficiency in preliminary in vivo experiments, and thus, they were not further tested to reduce the number of animals in use. The chitosan/kaolin nanocomposites resulted in higher survival rates among the tested animals, which can be explained by the faster time to hemostasis and lower amounts of blood loss and maintenance of a normal MAP, as compared to the QuikClot^®^ Combat Gauze. Both the chitosan/kaolin nanocomposites and QuikClot^®^ Combat Gauze were relatively more efficient in inducing hemostasis in comparison to the conventional dressing (Figure 2, Figure 3 and Figure 4). 

#### 2.2.2. In Vivo Hemostatic Efficiency in Rats

**Figure 5:**
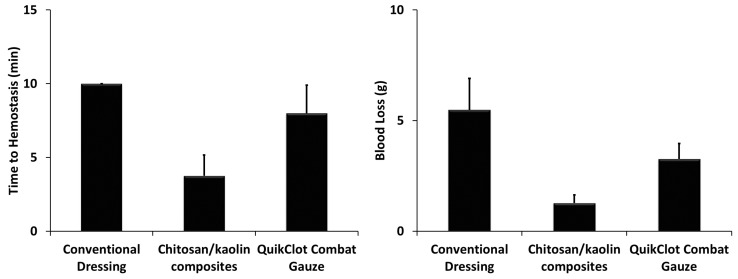
(**Left**): Total time to hemostasis in rats after cutting their femoral arteries and veins. The measurements were performed over 10 min and the animals that died in <10 min were assigned to a time to hemostasis of 10 min. The kaolin/chitosan composites resulted in significantly faster time to hemostasis compared to the conventional dressing (*p* < 0.0001) and QuikClot^®^ Combat Gauze (*p* < 0.001). The difference between conventional dressing and QuikClot^®^ Combat Gauze was not statistically significant. (**Right**): Total amounts of blood loss were evaluated by absorbing the leaked blood into conventional gauze, and the differences in the total weight of all the used pieces before and after the experiments were calculated. The kaolin/chitosan composites resulted in significantly lower amounts of blood loss compared to the conventional dressing (*p* < 0.0001) and QuikClot^®^ Combat Gauze (*p* < 0.01). The difference between conventional dressing and QuikClot^®^ Combat Gauze was also statistically significant (*p* < 0.01). In both experiments, conventional dressing, chitosan/kaolin composites, and QuikClot^®^ Combat Gauze were applied immediately after injury with immediate compression for 3 min.

The animal models were developed using the same procedures utilized in the rabbits, followed by immediate application of the hemostatic agents under pressure (weight of 200 g for 3 min), and measuring the total amount of blood loss and time to hemostasis. Similar to what has been observed in the severe injury model in rabbits, chitosan/kaolin composites resulted in faster time to hemostasis and lower amounts of blood loss, as compared to the QuikClot^®^ Combat Gauze. The latter was relatively more efficient in inducing hemostasis in comparison to the conventional dressing (Figure 5). 

#### 2.2.3. In Vivo Biocompatibility Examination in Rabbits

**Figure 6:**
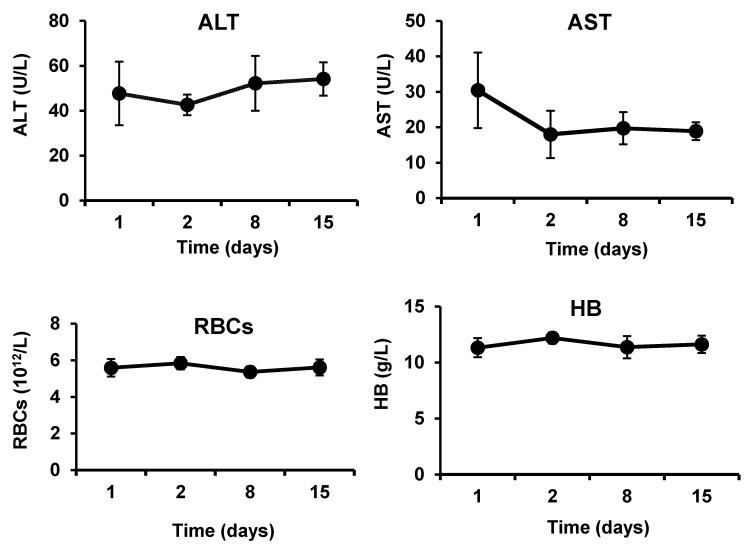

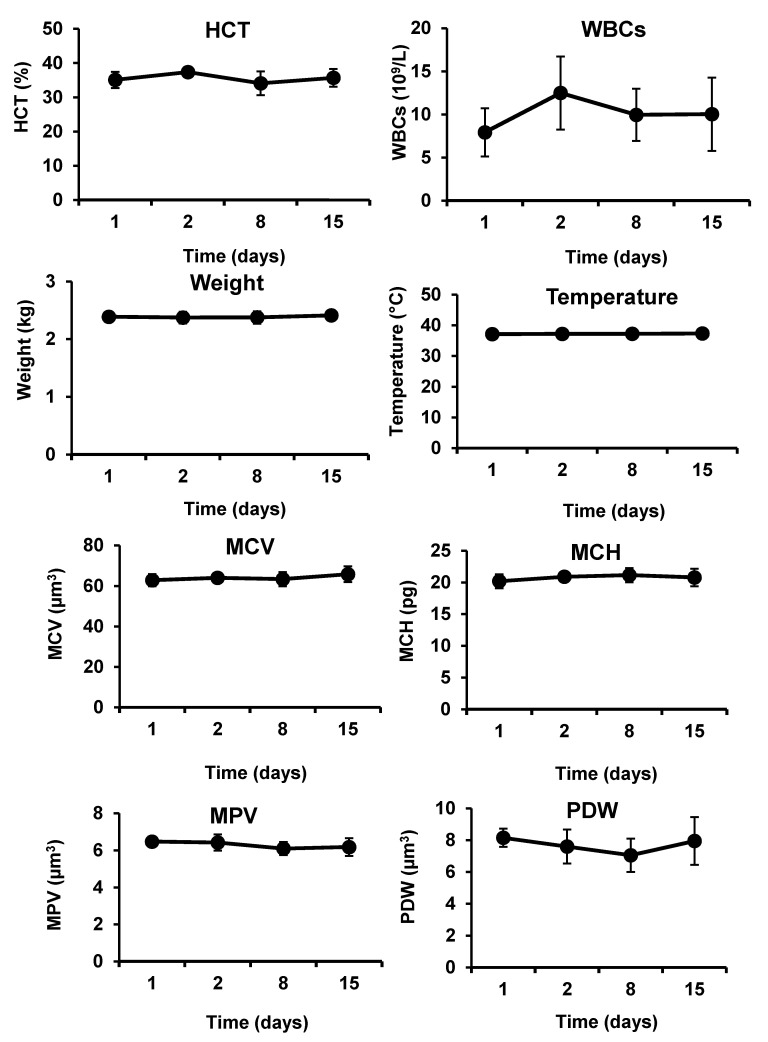
Biochemical changes in rabbits after cutting femoral artery, and applying chitosan/kaolin composite: Serum alanine aminotransferase (ALT) level, serum aspartate aminotransferase (AST) level, red blood cell (RBCs) counts, hemoglobin (HB) concentrations, hematocrit (HCT) percentages, white blood cell (WBCs) counts, body weights, body temperatures, mean corpuscular volume (MCV), mean corpuscular hemoglobin amounts (MCH), mean platelet volume (MPV), and platelet distribution width (PDW) of rabbits before the beginning of operation (day 1) and at 1 day, 7 day, and 14 day after the operation. The data represent the means ± SD (*n* = 5).

The animal models were developed by cutting the femoral artery, and applying immediately chitosan/kaolin nanocomposites under pressure (weight of 200 g for 3 min). Five rabbits were maintained under general anesthesia during the surgery and during the application of the dressings. Before the beginning of operation (day 1) and at 1 day, 7 day and 14 day after the operation, blood samples were taken using the vein puncture to analyze the alanine aminotransferase (ALT) and aspartate aminotransferase (AST), which were considered as indicators of hepatocellular toxicity. Meanwhile, RBCs, hemoglobin (HB) and hematocrit (HCT) were considered as indicators of hemolysis. White blood cells (WBCs) count was considered as an indicator of inflammation. Furthermore, the following markers were examined to provide further information on safety and biocompatibility of the hemostatic composite: MCV (mean corpuscular volume that measures the average size of red blood cells), MCH (mean corpuscular hemoglobin that refers to the average amount of hemoglobin found in the red blood cells), MPV (mean platelet volume that calculates the average size of platelets found in blood) and PDW (platelet distribution width that reflects how uniform the platelets are in size, i.e., a normal PDW indicates platelets that are mostly the same size, while a high PDW means that platelet size varies greatly, a clue that there is a platelet activation). After blood sampling, the weight and body temperature of animals were recorded. Values are presented as the means ± SD of five independent experiments. It can be clearly seen that the application of the chitosan/kaolin composites was safe and biocompatible (Figure 6). No changes in the liver enzymes (ALT and AST) were observed. The blood pictures have also demonstrated that the counts of RBCs and WBCs, and hemoglobin contents, were not affected by the application of the hemostatic composites. Furthermore, the mean corpuscular volume, mean corpuscular hemoglobin amount, mean platelet volume and platelet distribution width, were not altered. The animals also did not show signs of fever or weight loss. 

#### 2.2.4. In Vivo Biocompatibility Examination in Rats

**Figure 7:**
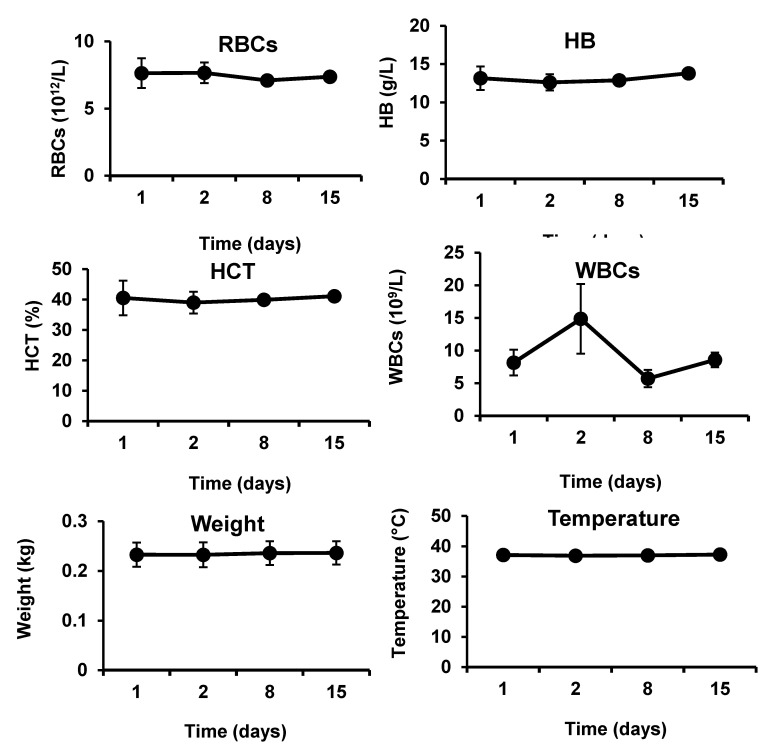

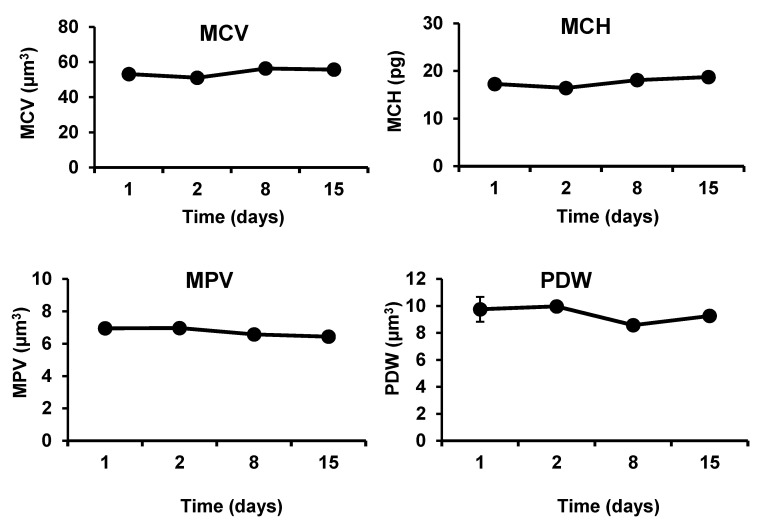
Clinical changes in rats after cutting femoral artery, and applying chitosan/kaolin composite: Red blood cell (RBCs) counts, hemoglobin (HB) concentrations, hematocrit (HCT) percentages, white blood cell (WBCs) counts, body weights, body temperature, mean corpuscular volume (MCV), mean corpuscular hemoglobin amounts (MCH), platelet distribution width (PDW), and mean platelet volume (MPV) of rats before the beginning of operation (day 1) and at 1 day, 7 day, and 14 day after the operation. The data represent the means ± SD (*n* = 5).

The animal models (five rats) were developed by utilizing the same procedures that have been described previously in the rabbits, followed by immediate application of the hemostatic composites under pressure (weight of 200 g for 3 min). Before the beginning of operation (day 1), and at 1 day, 7 day, and 14 day after the operation, blood samples were taken using the vein puncture to assess safety and biocompatibility of the composites in rats, as described in the previous section (vide supra). Similar to what has been observed in rabbits, application of the chitosan/kaolin composites was safe and biocompatible in rats (Figure 7). 

## 3. Discussion

Hemorrhage is one of the leading causes of death, globally. Induction of efficient hemostasis is of pivotal importance to control acute hemorrhage until the subjects receive the necessary medical care in the hospital. Biocompatible dressings of controlled surface properties that can induce quick hemostasis are currently under development in several laboratories [19,20,21,22,23]. In the current study, we have utilized two approaches to improve the hemostatic efficiency of the designed dressings. First approach is based on a combination between two commonly utilized hemostatic agents, kaolin and chitosan, to achieve a synergistic hemostatic effect. Second approach involved the use of Pluronic nanoparticles, or fibers on the micro- or nano-scale, to improve coating efficiency onto the carrier and interactions with biomolecules in the bleeding sites upon contact.

Similar to QuikClot^®^ Combat Gauze, the developed composites contain kaolin that meets USP specifications. Kaolin is an inert naturally occurring hydrated aluminum silicate mineral derived from clay that is widely used as a hemostatic agent. When exposed to plasma, kaolin activates clotting factor XII, a protein factor which assists in the initiation of the coagulation cascade [16,24]. It was reported in one study that addition of kaolin coating materials to blood of patients on anticoagulant therapy significantly reduced the time to clot formation (in vitro testing) [25]. The mean initial clot formation time was decreased from 692 s in the control to 190.8 s in the treated group (*p* < 0.0001), thus highlighting the ability of kaolin to reduce time to hemostasis and subsequently the amount of blood loss. However, efficient coating of kaolin onto the carrier is challenging. The use of heated kaolin slurry in coating the non-woven gauze is the most commonly utilized method for preparation of hemostatic dressings [16,17,18]. However, this method results in low coating efficiency, which explains the relatively low hemostatic competence of this kind of preparations [26]. 

The composites also contain chitosan, similar to Chitoflex^®^ and Celox™ Gauze. Chitosan is an organic polysaccharide carrying positively charged ions, which attracts negatively charged particles in blood, i.e., erythrocytes, to the wound sites and forms strong blood clots. The blood clots aggregating on the open wound result in hemostasis. Chitosan also has antibacterial property against certain bacterial strains [12,13]. Being manufactured from natural substance, chitosan wound dressing is biocompatible and biodegradable. 

The use of chitosan micro- or nano-based fibers resulted in high coating efficiency onto the carrier (Figure 1). As can be seen on the figure, deposition of chitosan onto the gauze resulted in spontaneous formation of microfibers of chitosan. However, formation of chitosan nanofibers is usually performed using other techniques. Electrospinning is one of the most commonly used strategies utilized for producing chitosan nanofibers [27,28]. Factors controlled to obtain fibers on the nanoscale include, blending with other polymers (i.e., PVA), molecular weight and concentration of chitosan, homogeneity and viscosity of chitosan solution, applied electric field, beading rate, and the distance between collector and needle of the electrospinning instrument [29]. Electron microscopy was then utilized to measure the average dimensions of the formed micro- and nano-nanofibers, which have demonstrated diameters of 2.4 ± 0.9 µm and 101 ± 56 nm, respectively (Figure 1).

Pluronics^®^ F-127 was also utilized to form nanoparticles that stabilize the hemostatic agent (i.e., kaolin) to increase stability, enhance disbursement, and to increase the surface area of the hemostatic agent, and/or the surface area covered or coated by the hemostatic agent (Figure 1). Pluronics are pharmaceutically acceptable and preferably approved by the United States Food and Drug Administration for human administration. Composites were prepared through impregnation of the carrier in a solution of the hemostatic composition and other ingredients (e.g., glycerin). After preparation, hemostatic devices were dried in air overnight, sterilized using autoclave, and further stored at room temperature till use. The pH of the prepared dressing after incubation in saline was in the range of 6–8, depending on the hemostatic agents utilized and additives presented in the dressing. When tested in vivo, the chitosan/kaolin composites resulted in exceptionally higher hemostatic performance in two animal models, rabbits and rats (Figure 2, Figure 3, Figure 4 and Figure 5).

This translation research is a clinical need-based to improve the hemostatic efficiency of the currently available commercialized hemostatic dressing via integration of more than one hemostatic agent, while utilizing nanotechnology-based approached to enhance the effective surface area of the hemostatic agents. Hence, the main focus was the in vivo screening to reach the optimized composites that could achieve exceptional hemostasis compared to the commercially available hemostatic agents.

In rabbits, time to hemostasis after applying the composites was 4.9 ± 1.6 min compared to 26.1 ± 22.8 min and 55.1 ± 13.8 min, for the QuikClot^®^ Combat Gauze and conventional dressing, respectively (Figure 3). The kaolin/chitosan composites resulted in significantly faster time to hemostasis compared to the conventional dressing (*p* < 0.0001) and QuikClot^®^ Combat Gauze (*p* < 0.05). The difference between conventional dressing and QuikClot^®^ Combat Gauze was also statistically significant (*p* < 0.01). The amount of blood loss after applying the composites was 3.5 ± 1.9 g compared to 25.1 ± 6.1 g and 31.1 ± 9.7 g for the QuikClot^®^ Combat Gauze and conventional dressing, respectively (Figure 3). The kaolin/chitosan composites resulted in significantly lower amounts of blood loss compared to the conventional dressing (*p* < 0.0001) and QuikClot^®^ Combat Gauze (*p* < 0.0001). The difference between conventional dressing and QuikClot^®^ Combat Gauze was not statistically significant. Percentages of survival recorded 1 and 24 h after applying the composites were 100% and 66.7% compared to 28.6% and 14.3%, and 33.3% and 0%, for the QuikClot^®^ Combat Gauze and conventional dressing, respectively (Figure 2). The kaolin/chitosan composites resulted in significantly longer survival duration compared to the conventional dressing (*p* < 0.01) and QuikClot^®^ Combat Gauze (*p* < 0.05). The difference between the conventional dressing and QuikClot^®^ Combat Gauze was not statistically significant. 

In rats, time to hemostasis after applying the composites was 3.8 ± 1.4 min compared to 8 ± 1.9 min and >10 min, for the QuikClot^®^ Combat Gauze and conventional dressing, respectively (Figure 5). The kaolin/chitosan composites resulted in significantly faster time to hemostasis compared to the conventional dressing (*p* < 0.0001) and QuikClot^®^ Combat Gauze (*p* < 0.001). The difference between conventional dressing and QuikClot^®^ Combat Gauze was not statistically significant. The amount of blood loss after applying the composites was 1.3 ± 0.4 g compared to 3.3 ± 0.7 g and 5.5 ± 1.4 g for the QuikClot^®^ Combat Gauze and conventional dressing, respectively (Figure 5). The kaolin/chitosan composites resulted in significantly lower amounts of blood loss compared to the conventional dressing (*p* < 0.0001) and QuikClot^®^ Combat Gauze (*p* < 0.01). The difference between conventional dressing and QuikClot^®^ Combat Gauze was also statistically significant (*p* < 0.01). 

To date, combination between more than one hemostatic agent has been tested in few studies. For instance, chitosan-based nonwoven dressings combined with recombinant batroxobin were tested in a rat femoral artery hemorrhage model [22]. Inclusion of recombinant batroxobin with chitosan resulted in a synergetic effect as illustrated by the improved blood coagulation and control of bleeding. In our study, the results clearly demonstrate the exceptional hemostatic efficiency of the kaolin/chitosan composites, in terms of reducing time to hemostasis and amounts of blood loss, when compared to QuikClot^®^ and conventional gauze. The differences observed between the QuikClot^®^ and conventional gauze in terms of time to hemostasis and amounts of blood loss were not always statistically significant. Won-Suk Lee and coworkers have conducted a retrospective study to compare QuikClot^®^ and conventional gauze during elective laparoscopic colorectal surgery in 200 patients (100 patients per group) [26]. Time to hemostasis after the two types of treatments was evaluated. The mean time to hemostasis in both groups was 3.5 min vs. 4.4 min (*p* = 0.584), thus implicating no statistically significant difference between the two groups, and thus QuikClot^®^ application to the surgery sites did not provide additional benefits to the patients compared to the conventional gauze. The data from this study are in complete agreement with what we have observed in the two animal models, rabbits and rats. 

The developed hemostatic composites demonstrated in vivo biocompatibility in severe injury models in both rabbits and rats (Figure 6 and Figure 7). The levels of ALT and AST were not significantly altered after application of the nanocomposites to the femoral artery injury sites in rabbits. Counts of RBCS and WBCs, hemoglobin contents, blood cells, and platelets characteristics were not also significantly changed after exposure of the injury sites to the hemostatic composites, in rats and rabbits. No signs of weight loss or fever have been observed in the animals throughout the experiments. Absence of clinical and biochemical changes (i.e., hepatocellular toxicity, hemolysis, inflammation, and platelets activation) following application of the chitosan/kaolin composites demonstrates the biocompatibility of the composites. The observed biocompatibility is in agreement with previously reported results from our group and others [14,30,31,32]. For instance, Won-Suk Lee and coworkers have reported on safety and biocompatibility of QuikClot^®^ and conventional gauze during elective laparoscopic colorectal surgery in 200 patients (100 patients per group) [26]. Hemoglobin and hematocrit levels were similar in the two groups when assessed 3 d after surgery in the Jackson–Pratt drain (i.e., post-operative drains from the surgical sites). Both types of dressings were biocompatible with no reported adverse reactions in both groups. 

The developed composites aim at achieving prompt and efficient hemostasis via increasing the specific surface area of hemostatic agents in order to increase the coating efficiency onto the carrier (i.e., gauze) and to enhance the contact with the body tissues and blood cells. The developed composites include additional features that contributed to the improved hemostatic efficiency of the developed dressing. These features include (1) combination of two hemostatic agents (kaolin and chitosan) that act via different hemostatic mechanisms, and (2) the use of nanotechnology-based approaches to allow deposition of hemostatic agents onto nanoscale features of greater surface areas. 

In conclusion, in two animal models, namely rabbits and rats, at the sites of femoral artery and vein injuries, hemostasis has been achieved upon applying the chitosan/kaolin nanocomposites, whereas higher amounts of blood loss and continuous bleeding could be seen at the sites of injuries after utilizing the conventional dressing and the QuikClot^®^ Combat Gauze, although all the experimental factors were fixed except for the type of the dressing. The nanocomposites have also demonstrated safety and biocompatibility, as demonstrated by blood pictures and the absence of signs of inflammation, hepatocellular toxicity and physical changes. The significant improvement in hemostatic efficiency and high safety and biocompatibility profiles illustrated in the animal models highlight the auspicious potential of the developed hemostatic composites in controlling bleeding from acute hemorrhage. 

## 4. Materials and Methods

### 4.1. Preparation of Chitosan/Kaolin Hemostatic Composites

Non-woven gauze impregnated with kaolin and chitosan, glycerin and Pluronic^®^ F-127 was prepared. Chitosan of high-molecular weight (310,000–375,000 Da, >75% deacetylation degree) was used as acetate salts via dissolving chitosan in acetic acid. The total amount of the hemostatic agents was ca. 50% of the total weight of the dressing, whereas Pluronic was ca. 1% of the total weight. Total impregnation time was 5 min. Pluronic^®^ F-127 nanoparticles were prepared in water and stirred (hotplate stirrer, Daihan Scientific Co., Ltd., Seoul, South Korea) for 2 h prior to the impregnation step, and were utilized to stabilize the prepared kaolin. Hemostatic agents (e.g., kaolin and chitosan) were added gradually to the previously formed solution under stirring and heating. The coated gauze substrate was then rolled to further embed the hemostatic agents into the material of the substrate. The gauze has been rolled under high pressure, and the dust has been removed via air nozzles. Both the slurry and the final dressings were sterilized in the autoclave.

To prepare the chitosan nanofibers, chitosan solution (3 wt.%) was prepared in 20 mL of 70% glacial acetic acid under stirring at 50 °C for 2 h, followed by continuous stirring at room temperature overnight (14 h). Polyvinyl alcohol (PVA, molecular weight 115,000 g/mole, degree of polymerization 1700–1800) solution (10%) was prepared in 20 mL distilled water, stirred at 80 °C for 2 h, followed by continuous stirring at room temperature overnight (14 h). Chitosan/PVA solution was prepared at a ratio of 50/50, followed by stirring at the room temperature, and then the solution was sonicated for 2 h. For electrospinning, the chitosan/PVA solution was withdrawn into a 10 mL plastic syringe connected to a stainless-steel needle of 0.8 mm diameter. The syringe was placed in a syringe pump and the needle was connected to a high voltage supply. Preferred conditions to get chitosan nanofibers were: High molecular weight chitosan, 3% chitosan solution, 10% PVA solution, applied voltage ca. 20 kV, flow rate ca. 0.4 mL/h, distance between needle tip and the collector is 10 cm. Chitosan/PVA nanofibers were dried at 60 °C for 2 h, and then stored in a laboratory desiccator until characterization. 

The type of chitosan is deacetylated chitin with a degree of deacetylation of ca. 75%. The source of chitosan is from shrimp shells. Chitosan was used after dissolution in 1% acetic acid under stirring overnight, followed by neutralization with 0.1 N NaOH. The carrier was impregnated separately into the chitosan solution. After drying, it was immersed into the kaolin slurry. The mixture of chitosan and kaolin was homogenous as demonstrated by visual inspection and optical imaging (data not shown). Mixing of the kaolin and chitosan has been also accomplished where they form homogenous dispersion, with no agglomeration or precipitation. It has been reported previously that there are strong interactions between chitosan and kaolin as revealed by FTIR and thermogravimetric analysis [33]. It was found previously by SEM that the kaolin particles are well dispersed in the chitosan matrix [33]. 

### 4.2. Characterizations of Chitosan/Kaolin Hemostatic Composites

The surface morphologies of the electrospun chitosan/PVA nanofiber and chitosan microfibers were studied by using field emission-scanning electron microscope (FE-SEM, Quanta FEG 250, Field Electron and Ion Company, Hillsboro, OR, USA) at an accelerating voltage of 30 kV. Samples were fixed onto a holder with the aid of carbon tape and then placed in the sputtering machine for platinum coating, to increase their electrical conductivity. After platinum coating, the microfibers and electrospun nanofibers were examined by FE-SEM under high vacuum. Average fiber diameters were determined by measuring fibers that were randomly selected from SEM images using Image J software (1.53e, National Institutes of Health, Bethesda, MD, USA).

For measurement of the pH, area of 5 cm^2^ of the dressing was placed into 50 mL of saline. The pH was measured after 2 h. The pH values were in the range of 6–8.

### 4.3. In Vivo Hemostatic Efficiency in Rabbits and Rats

Twenty-seven adult rabbits (males and females, ca. 2 kg) were purchased locally and kept in large cages at the room temperature and allowed food and water ad libitum. Rabbits were anesthetized with intraperitoneal injection of urethane (1 g kg^−1^) prior to the surgical procedures. Rabbits were randomly selected from the laboratory population and divided into three groups (*n* = 9). The first group served as a control group which received the conventional dressing, the second group received the chitosan/kaolin composites, and the last group received the QuikClot^®^ Combat Gauze. Animals were maintained under general anesthesia throughout the experiments and euthanatized while under anesthesia at the end of the experiments. The animal models were developed by cutting the femoral artery and vein, and applying immediately the hemostatic agents under pressure (weight of 200 g for 3 min) and measuring the survival rates, survival durations, total amount of blood loss, time to hemostasis, and mean arterial blood pressure (MAP). No fluid resuscitation or surgical intervention was allowed during the experimental setup. The weights of the dressings were recorded before application and at the end of the experiments to measure the amounts of blood loss. MAP was recorded via insertion of a 20-gauge catheter into the right carotid artery and continuous recording of MAP under general anesthesia before the injury and over 1 h after the injury. Blood pressure was monitored on a polygraph using a Universal Oscillograph instrument (Harvard Apparatus, South Natick, MA, USA).

Eighteen adult male Sprague–Dawley rats (ca. 300 g) were anaesthetized with an intraperitoneal injection of sodium thiopental (50 mg kg^−1^) and their abdominal hair was shaved. The rats were randomly selected from the laboratory population and divided into three groups (*n* = 6). The animals were kept in large cages at the room temperature and allowed food and water ad libitum. The first group served as a control group which received the conventional dressing, the second group received the chitosan/kaolin dressing, and the last group received the QuikClot^®^ Combat Gauze. Animals were maintained under general anesthesia throughout the experiments and euthanatized while under anesthesia at the end of the experiments. The animal models were developed by cutting the femoral artery and vein, and applying immediately the hemostatic agents under pressure (weight of 200 g for 3 min) and measuring the total amount of blood loss and time to hemostasis. The weights of the dressings were recorded before application and at the end of the experiments to measure the amounts of blood loss. No fluid resuscitation or surgical intervention was allowed during the experimental setup.

### 4.4. In Vivo Biocompatibility in Rabbits and Rats

Five adult rabbits (males and females, ca. 2 kg) were purchased locally and kept in large cages at the room temperature and allowed food and water ad libitum. Rabbits were anesthetized with intraperitoneal injection of urethane (1 g kg^−1^) prior to the surgical procedures. Rabbits were randomly selected from the laboratory population (*n* = 5). The animals before the surgical procedures were considered as the control (day 1). The animal models were developed by cutting the femoral artery, and applying immediately chitosan/kaolin composites under pressure (weight of 200 g for 3 min). Animals were maintained under general anesthesia during the surgery and during the application of the dressings. Before the beginning of operation (day 1) and at 1 day, 7 day, and 14 day after the operation, blood samples were taken using the vein puncture to analyze the alanine aminotransferase (ALT) and aspartate aminotransferase (AST), which were considered as indicators of hepatocellular toxicity. Meanwhile, red blood cell (RBCs), hemoglobin (HB), and hematocrit (HCT) were considered as indicators of hemolysis, and white blood cell (WBCs) count was considered as an indicator of inflammation. Furthermore, the following markers were examined to provide further information on safety and biocompatibility of the hemostatic composite: MCV (mean corpuscular volume that measures the average size of red blood cells), MCH (mean corpuscular hemoglobin that refers to the average amount of hemoglobin found in the red blood cells), MPV (mean platelet volume that calculates the average size of platelets found in blood) and PDW (platelet distribution width that reflects how uniform the platelets are in size, i.e., a normal PDW indicates platelets that are mostly the same size, while a high PDW means that platelet size varies greatly, a clue that there is a platelet activation). After blood sampling, the weight and body temperature of animals were recorded. Values are presented as the mean ± SD of five independent experiments.

Five adult male Sprague–Dawley rats (ca. 300 g) were anaesthetized with an intraperitoneal injection of sodium thiopental (50 mg kg^−1^) and their abdominal hair was shaved. The rats were randomly selected from the laboratory population (*n* = 5) and were kept in large cages at the room temperature and allowed food and water ad libitum. The animals before the surgical procedures were considered as the control (day 1). The animal models were developed by cutting the femoral artery, and applying immediately the hemostatic composites under pressure (weight of 200 g for 3 min). Animals were maintained under general anesthesia during the surgery and during the application of the dressings. Before the beginning of operation (day 1), and at 1 day, 7 day, and 14 day after the operation, blood samples were taken using the vein puncture to assess safety and biocompatibility of the composites in rats, as described in the previous section (vide supra).

### 4.5. Statistical Analysis

Values are presented as the mean ± SD of at least five independent experiments. Significant differences between groups were evaluated by one-way ANOVA followed by Tukey’s multiple comparison tests. Differences between different groups were considered significant for *p* values less than 0.05. A sample size of nine rabbits per group, for time to hemostasis and blood loss experiments, was expected to provide a power of ca. 0.9, with a Type I error probability for rejection of the null hypothesis of 0.05. Then, fewer numbers of animals have been utilized for other experiments to reduce the number of animals utilized throughout the study.

## Data Availability

The data presented in this study are available on request from the corresponding author.
